# Assessment of public knowledge and perception of stroke, seizures, and subarachnoid hemorrhage among adults aged ≥18 years in Saudi Arabia: A cross-sectional study

**DOI:** 10.1097/MD.0000000000047192

**Published:** 2026-01-16

**Authors:** Shorog Althubait, Taif Ali A. Almazni, Ghaida Mohammed Al Hunaif, Layan Saeed Alshmrani, Wrod Ahmed S. Alqahtani, Mohammed Abdullah Alzayd, Mohammed Moteb M. Alshahrani, Shahad Ali M. Alshehri, Saud Turki M. Alqahtani, Layan Dulaym Dashnan, Syed Esam Mahmood

**Affiliations:** a Neurology, Department of Internal Medicine, College of Medicine, King Khalid University, Abha, Saudi Arabia; b College of Medicine, King Khalid University, Abha, Saudi Arabia; c Department of Family and Community Medicine, College of Medicine, King Khalid University, Abha, Saudi Arabia.

**Keywords:** cross-sectional study, health literacy, neurological emergencies, public health knowledge, Saudi Arabia, seizure first aid, stroke awareness, symptom recognition

## Abstract

Neurological emergencies, including stroke, seizures, and subarachnoid hemorrhage, require prompt recognition and intervention for optimal outcomes. Public awareness of these conditions is essential, yet knowledge gaps persist in Saudi Arabia despite their increasing prevalence. This study aims to assess knowledge and perceptions of neurological emergencies among the Saudi general population and identify sociodemographic factors influencing awareness levels. A cross-sectional study was conducted between March and July 2025 using an online questionnaire distributed to Saudi residents aged ≥18 years. The survey assessed knowledge of symptoms, appropriate responses, and sources of information regarding neurological emergencies. Data were analyzed using SPSS version 29, with nonparametric tests to evaluate differences between demographic groups. Among 593 participants, 60.7% correctly identified initial actions for stroke, while 80.6% recognized that even one stroke symptom warrants immediate action. Most participants knew not to perform cardiopulmonary resuscitation if breathing (91.2%) or sprinkle water (85.3%), but fewer identified proper actions like turning the person on their side (44.9%) or calling emergency services for seizures over 5 minutes (50.8%). Gaps exist in recognizing emergency situations such as injuries or prolonged unconsciousness. Female participants, residents of Central and Western regions, and those with healthcare backgrounds demonstrated significantly higher knowledge scores (*P* < .05). Social media (62.6%) was the primary information source, followed by healthcare professionals (55.3%). This study revealed notable gaps in public knowledge about neurological emergencies in Saudi Arabia, particularly regarding recognition of stroke symptoms and appropriate seizure management. However, over 80% of participants were aged 18 to 30, which limits generalizability to the broader Saudi population. Findings highlight the need for targeted educational interventions, especially for demographics showing lower awareness levels, to improve early recognition and management to these time-sensitive conditions.

## 1. Introduction

Neurological disorders, such as stroke, seizures, and subarachnoid hemorrhage (SAH), are among the leading causes of disability worldwide and rank as the second leading cause of death.^[1]^ Stroke alone remains a major contributor to long-term disability and mortality globally.^[2]^ The rapid identification and treatment of these emergencies are critically important because timely intervention can significantly reduce brain damage, improve survival rates, and enhance long-term functional outcomes.^[3]^ For instance, early administration of thrombolytic therapy in ischemic stroke patients can restore blood flow and minimize neurological deficits, but its effectiveness diminishes rapidly with time (highlighting the “time is brain” principle). Similarly, prompt recognition of seizures can prevent injury and complications, while rapid diagnosis of SAH is essential to prevent rebleeding and secondary brain injury.

This urgency is especially pronounced in regions like the Middle East, including Saudi Arabia, where the incidence of stroke and other neurological emergencies is increasing due to demographic shifts, lifestyle changes, and rising prevalence of risk factors such as hypertension and diabetes.^[4]^ Delays in diagnosis often lead to worse outcomes, increased healthcare costs, and greater societal burden through disability and loss of productivity.

Public awareness of neurological emergency symptoms is vital for early intervention and improved prognosis. However, evidence from both international and regional studies reveals widespread knowledge gaps and misconceptions.^[5–7]^ Many individuals are unable to recognize common warning signs of stroke, such as sudden weakness, speech difficulties, or facial droop, leading to delays in seeking emergency care. In a recent survey of Arabic-speaking adults, only about 25% could identify all early stroke symptoms.^[3]^ Misconceptions about seizures are also common, including beliefs in supernatural causes and unsafe first-aid practices such as inserting objects into the mouth during a seizure.^[8]^

In Saudi Arabia, community studies report low seizure first-aid awareness, with average scores around 57%, and limited familiarity with appropriate actions during active seizures.^[9]^ These deficits are further compounded by myths, including false beliefs that epilepsy is contagious or psychological.^[8]^ Sociodemographic factors such as age, gender, and education significantly influence awareness; generally, younger, more educated individuals or those with prior exposure to neurological conditions display better knowledge.^[3]^ Sources of health information, including awareness campaigns, healthcare providers, and social media, play critical roles in disseminating accurate knowledge.^[9]^ Despite some progress, the persistence of misconceptions and low awareness underscore the urgent need for targeted educational interventions.

In the context of Saudi Arabia, where the prevalence of stroke and other neurological emergencies is rising, understanding public perceptions and recognition of these conditions is essential for effective emergency response. Currently, there is a paucity of comprehensive data on how well the Saudi population recognizes stroke, seizures, SAH, and acute headaches, as well as the immediate actions they would take. This knowledge gap hampers efforts to develop effective public health strategies aimed at early detection and prompt treatment.

This study aims to assess knowledge and perceptions of neurological emergencies among Saudi adults and to explore how sociodemographic variables such as age, gender, education, and region influence awareness. The findings are intended to inform targeted public health initiatives and educational strategies that improve early recognition and response, ultimately reducing preventable morbidity and mortality associated with these life-threatening conditions.

## 2. Methods

### 2.1. Study design

This study utilized a cross-sectional survey design to assess public awareness and understanding of neurological emergencies in Saudi Arabia. It was conducted between March 2025 and July 2025. Data were collected through structured online questionnaires, ensuring accessibility to a diverse population across different socioeconomic backgrounds, age groups, and educational levels.

### 2.2. Study population

Participants were recruited using convenience and snowball sampling methods. The target participants included individuals aged 18 years and older who were residents of Saudi Arabia with internet access and the ability to complete an online questionnaire. Only willing participants who provided informed consent were included in the study, while those who did not meet these criteria were excluded.

The sample size was calculated according to the formula for determining proportion in a survey using the Raosoft online sample size calculator. With a margin of error of 5%, a confidence level of 95%, and the assumption that 50% of the people had adequate knowledge, the minimum required sample size was determined to be 385 people.

### 2.3. Data collection

The questionnaire was developed following a thorough review of existing literature on public awareness and response behaviors related to neurological emergencies. While no single instrument was exclusively relied upon, the design was guided by key findings from prior research, particularly studies focusing on stroke and epilepsy.^[10,11]^ The structure of the questionnaire was crafted to reflect current clinical understanding and to identify common knowledge gaps among the target population. Sections addressing SAH and acute headaches were formulated based on established clinical guidelines and general neurological knowledge. To ensure content validity and cultural appropriateness, the questionnaire was developed through an expert consensus process involving neurologists and public health specialists. Their contributions helped shape the phrasing, relevance, and comprehensiveness of the items, ensuring the instrument accurately reflects essential knowledge and perceptions regarding these conditions.

The final instrument comprised 5 sections: demographics; general awareness of neurological emergencies; stroke knowledge; seizure knowledge; and awareness of SAH and headaches. To ensure broad applicability and cultural relevance, the questionnaire was distributed online to a diverse sample of individuals across Saudi Arabia. Participation was voluntary and anonymous, with an estimated completion time of 5 to 10 minutes. Participants were informed that they could withdraw at any point without penalty.

Prior to the main data collection, a pilot study was conducted to evaluate the feasibility, clarity, and accessibility of the online survey. Each collaborator was asked to provide at least 2 responses to assess the questionnaire’s functionality. The pilot included a cover page explaining the study’s purpose, detailed instructions, and a consent form. The feedback obtained helped refine the survey and provided estimates of response rates and completion times, ensuring the instrument’s practicality for the larger study.

### 2.4. Statistical analysis

The statistical analysis was done using SPSS version 29 for Windows (SPSS Inc., Chicago). The items measuring the awareness level were sorted and tested for reliability using Cronbach alpha. After exclusion of 5 items, the reliability score was (*R* = 0.82). The total then was calculated out of 29 items. Continuous variables were summarized using median and interquartile range after applying the normality tests Kolmogorov–Smirnov and Shapiro–Wilk tests. Categorical variables were presented using frequency tables and proportions. Differences between groups in terms of awareness levels were assessed using non-parametrical tests, Mann–Whitney for binary groups, and Kruskal–Wallis for variables with >2 groups. *P*-values <.05 were considered to be significant.

### 2.5. Ethical considerations

The study only enrolled participants who provided consent before completing the questionnaire, and their data was accessible only to researchers. Ethical permission was obtained from the Ethics Committee of King Khalid University (ECM#2025-709), kept confidential, and participation in the study was completely voluntary.

## 3. Results

A total of 593 participants were included in this cross-sectional study. The majority of participants (80.6%) were between the ages of 18 to 30 years, with females constituting 66.1% of the sample. Most participants were Saudi nationals (98.0%), with 34.2% residing in the Southern region of Saudi Arabia. Regarding educational level, 80.9% held a university degree, while only 0.6% had primary school education or no formal education. The sample included medical field students (31.0%), nonmedical field students (30.2%), non-healthcare employees (17.5%), unemployed individuals (19.7%), and healthcare professionals (1.5%) (Table 1).

**Table 1:** Sociodemographic characteristics of study participants (N = 593).

Variable	Groups	N	%
Age groups	18–30	478	80.6%
	31–40	39	6.6%
	41–50	56	9.4%
	More than 50	20	3.4%
Gender	Female	392	66.1%
	Male	201	33.9%
Nationality	Saudi	581	98.0%
	Non-Saudi	12	2.0%
Region	Central region	118	19.9%
	East region	110	18.5%
	North region	66	11.1%
	West region	96	16.2%
	South region	203	34.2%
Educational level	No formal education	2	0.3%
	Primary school	2	0.3%
	High school	95	16.0%
	University degree	480	80.9%
	Postgraduate degree	14	2.4%
Occupation	Medical field student	184	31.0%
	Nonmedical field student	179	30.2%
	Health care professional	9	1.5%
	Non-healthcare employee	104	17.5%
	Unemployed	117	19.7%

Regarding sources of information and personal experience with neurological emergencies, 27.5% of participants reported having personal or family experience with neurological emergencies such as stroke, seizure, or SAH. Social media platforms (62.6%) were the most common source of information about neurological emergencies, followed by healthcare professionals (55.3%), family and friends (37.6%), books (29.2%), and TV/news channels (24.6%). Only 0.8% of participants reported obtaining information from other sources (Table 2).

**Table 2:** Sources of information and personal experience regarding neurological emergencies among study participants (N = 593).

Variable	Yes		No	
	N	%	N	%
Have you or someone in your family ever experienced a neurological emergency (stroke, seizure, or SAH)?	163	27.5%	430	72.5%
*Source of information*				
Books	173	29.2%	420	70.8%
Doctors/healthcare professionals	328	55.3%	265	44.7%
Social media (X, Instagram, TikTok, etc)	371	62.6%	222	37.4%
TV/news channels	146	24.6%	447	75.4%
Family and friends	223	37.6%	370	62.4%
Other	5	0.8%	588	99.2%

SAH = subarachnoid hemorrhage.

When assessing participants’ knowledge and response to neurological emergencies, 60.7% correctly identified the first action to take if someone was having a stroke. Most participants (80.6%) accurately recognized that even one stroke symptom warrants immediate action, and 53.3% knew that prompt treatment for stroke likely leads to better outcomes. For sudden severe headaches with vomiting and neck stiffness, 81.3% would take appropriate action. Regarding stroke symptom recognition, participants correctly identified focal weakness (52.8%) and mouth deviation (51.1%) as symptoms, while 70.8% correctly identified that chest pain is not a primary stroke symptom. However, only 47.7% recognized weakness in the arm or leg as a stroke symptom (Table 3).

**Table 3:** Participants knowledge and response to neurological emergencies questions (N = 593).

Statements	Correct answer		Wrong answer	
	N	%	N	%
If you thought someone was having a stroke, what is the first thing you would do?	360	60.7%	233	39.3%
How many stroke symptoms must a person have before you would do something?	478	80.6%	115	19.4%
If a person has a stroke and gets treatment quickly they will most likely	316	53.3%	277	46.7%
If you or someone else experiences a sudden, severe headache with vomiting and neck stiffness, what would you do?	482	81.3%	111	18.7%
*Symptoms of stroke*				
Mouth deviation	303	51.1%	290	48.9%
Focal weakness (dropping of face)	313	52.8%	280	47.2%
Chest pain	420	70.8%	173	29.2%
Weakness in the arm or leg	283	47.7%	310	52.3%
*What should you do if someone is having a seizure?*				
Sprinkle water on the person’s face to wake up	506	85.3%	87	14.7%
Clear the area around the person of anything hard or sharp	384	64.8%	209	35.2%
Stay by their side, and record the time and duration of the seizure	304	51.3%	289	48.7%
Neck Loosen tight clothing and remove any tight eyeglasses or chains	311	52.4%	282	47.6%
Turn the person gently onto one side if possible	266	44.9%	327	55.1%
Perform cardiopulmonary resuscitation (CPR), even if he or she is breathing	541	91.2%	52	8.8%
Give an epilepsy pill by mouth to stop the seizure	480	80.9%	113	19.1%
If the seizure ends, stay with them until they are fully awake and tell them what happened	338	57.0%	255	43.0%
*When should you call an ambulance for someone having a seizure?*				
You do not know the person	184	31.0%	409	69.0%
The person has never had a seizure before	261	44.0%	332	56.0%
The seizure lasts for 5 min or more	301	50.8%	292	49.2%
Another seizure begins soon after the first one	258	43.5%	335	56.5%
The person does not regain consciousness within 5 min	282	47.6%	311	52.4%
The person has difficulty breathing after the seizure	270	45.5%	323	54.5%
The person was seriously injured during the seizure	282	47.6%	311	52.4%
The person has a health condition like diabetes or heart disease	227	38.3%	366	61.7%
*Which of the following do you believe can indicate a serious neurological emergency related to headaches?*				
Sudden, severe headache (worst headache of life)	408	68.8%	185	31.2%
Gradual, mild headache that goes away with rest	525	88.5%	68	11.5%
Headache accompanied by neck stiffness and vomiting	360	60.7%	233	39.3%
Headache that worsens with exertion or changes in position	194	32.7%	399	67.3%
Headache after head trauma	307	51.8%	286	48.2%

Concerning seizure management, most participants correctly identified inappropriate actions: 91.2% knew not to perform cardiopulmonary resuscitation if the person is breathing, 85.3% recognized that sprinkling water on the face is inappropriate, and 80.9% knew not to administer oral medication during a seizure. Appropriate interventions were less consistently identified: 64.8% would clear the area of hard or sharp objects, 57.0% would stay with the person until fully awake, 52.4% would loosen tight clothing, 51.3% would stay by their side and record the seizure duration, and only 44.9% would turn the person onto their side. Regarding when to call an ambulance for seizures, only 50.8% correctly identified that seizures lasting 5 minutes or more warrant emergency services. Less than half recognized other critical situations requiring emergency response: breathing difficulties (45.5%), serious injuries (47.6%), prolonged unconsciousness (47.6%), first-time seizures (44.0%), multiple seizures (43.5%), or seizures in unknown individuals (31.0%) (Table 3).

Analysis of neurological emergencies awareness revealed a median score of 16.00 (interquartile range: 12.00–21.00), with minimum and maximum scores of 5.00 and 29.00, respectively. Analysis of factors associated with knowledge of neurological emergencies revealed significant variations across demographic characteristics. Gender was a significant factor, with females demonstrating higher knowledge scores compared to males (*P* < .05). Regional differences were also significant, with participants from the Central and Western regions showing higher mean ranks than those from other regions (*P* < .001). Occupation also emerged as a significant factor, with healthcare professionals and medical field students displaying the highest mean ranks (317 and 334, respectively), indicating superior knowledge compared to other occupational groups (*P* < .001). Educational level, age, and nationality showed no statistically significant association with neurological emergency knowledge levels (*P* > .05). Results are shown in (Table 4).

**Table 4:** Comparison of neurological emergency knowledge scores across demographic variables among study participants (N = 593).

Variables	Groups	Mean rank	*P*-value
Age groups	18–30	302.9	**.053**
	31–40	230.09	
	41–50	306.71	
	More than 50	259.3	
Gender	Female	319.28	**<.001**
	Male	253.55	
Nationality	Saudi	295.08	**.057**
	Non-Saudi	390.04	
Region	Central region	321.39	**.048**
	East region	305.13	
	North region	245.88	
	West region	310.43	
	South region	288.68	
Educational level	No formal education	325.25	.407
	Primary school	450.75	
	High school	315.62	
	University degree	293.92	
	Postgraduate degree	250.32	
Occupation	Medical field student	333.58	**.010**
	Nonmedical field student	286.36	
	Health care professional	317	
	Non-healthcare employee	269.23	
	Unemployed	278.89	

Bold value indicates statistically significant result at the .05 level.

## 4. Discussion

Neurological emergencies such as stroke, seizures, and SAH require immediate recognition and response to prevent severe outcomes. This study assessed public awareness of these critical conditions among the Saudi population and revealed notable gaps. While many participants recognized stroke as an emergency, knowledge of specific warning signs and appropriate seizure first aid was often limited. Enhancing public awareness is essential because delayed symptom recognition can lead to treatment delays and worse patient outcomes.^[12]^

Overall, the public awareness of neurological emergencies in our study was moderate but suboptimal. Although the majority acknowledged stroke as a serious condition, detailed symptom recognition was less robust. Approximately two-thirds of respondents could identify common stroke symptoms such as speech difficulty or one-sided weakness, consistent with findings from regional and international studies that report 30% to 50% of people cannot name all stroke warning signs.^[12,13]^ Significant gaps were found in seizure awareness; fewer respondents knew proper seizure first aid.

Our results mirror a national Saudi survey reporting an average 57% knowledge score on seizure first aid, with many still unaware that placing objects in the mouth during a seizure is harmful, a widespread misconception.^[9]^ Female participants and those with medical backgrounds scored higher, possibly reflecting women’s greater engagement with health information and caregiving roles.^[14]^ Regional differences in awareness were also apparent, with participants from urban, resource-rich areas showing higher knowledge than those in rural settings. Such disparities may be due to differences in education, health literacy, access to information, and cultural beliefs.^[15,16]^ Notably, a minority still attributed epilepsy to supernatural causes, which hinders factual understanding. These findings highlight uneven but improvable public awareness influenced by sociodemographic and regional factors.^[17]^

Our findings align with multiple studies from Saudi Arabia and the broader Middle East, which also demonstrate moderate awareness coupled with critical knowledge gaps. For example, a 2022 survey in Taif found that 61.7% of adults had some stroke knowledge but struggled to recognize specific stroke signs.^[18]^ Similarly, research in Saudi Arabia’s Western region described stroke awareness as only moderate, with many unable to identify all symptoms. These parallels indicate that although basic awareness of stroke as an emergency is common, detailed understanding remains inadequate across Saudi communities.^[19]^ Regional epilepsy studies corroborate our seizure findings: only half the public knew key first-aid measures, with misconceptions such as attempting to insert objects during seizures persisting.^[9]^ Our observation that women are generally more knowledgeable than men matches regional data showing female respondents more frequently recognize epilepsy facts and reject stigma.^[17]^ However, cultural beliefs and stigma regarding epilepsy remain entrenched in some communities, reflecting historical attitudes in the region.^[17]^

Encouragingly, public health campaigns by Saudi authorities over the past decade have modestly improved stroke and epilepsy recognition in urban areas. Nevertheless, rural and underserved populations continue to show the lowest awareness levels, a pattern echoed by studies from Egypt and other Middle Eastern countries.^[15]^ Factors such as education quality, healthcare infrastructure, and local outreach efforts contribute to this divide. Countries with stronger healthcare education systems, such as Jordan and Gulf states, report slightly better public knowledge, while conflict-affected or resource-limited nations show poorer awareness.^[20]^ Overall, our study fits within a regional context of improving but still insufficient public knowledge, underscoring the need for sustained, culturally tailored awareness initiatives.

Globally, public awareness of stroke and other neurological emergencies varies but faces similar challenges. In high-income countries, knowledge has improved due to public campaigns, yet gaps remain. In the United States, surveys from the mid-2010s showed about two-thirds of adults recognized major stroke symptoms, with women generally more aware than men.^[14,21]^ However, only 16% felt knowledgeable about epilepsy, and just 25% knew proper seizure first aid, a pattern closely resembling our findings.^[22]^ European nations report suboptimal stroke literacy; a multicountry survey found nearly 20% could not name a single stroke symptom, and less than half would call an ambulance during a stroke emergency.^[23]^

UK campaigns like “Act FAST” have temporarily boosted recognition and emergency calls, but sustaining these gains remains challenging.^[24]^ In Asia, awareness levels are often lower, especially in rural areas. Studies in Malaysia showed only around 36% could identify all stroke symptoms, and less than half knew to call emergency services.^[25]^ Large Chinese surveys found poor recognition of less obvious stroke signs, contributing to delays.^[12]^ In low- and middle-income countries such as Uganda, most people lack basic stroke knowledge, and myths around epilepsy prevail, including harmful traditional practices.^[26]^ Despite healthcare system and cultural differences, a universal challenge emerges: closing the knowledge gap through effective education. Misconceptions such as placing objects in the mouth during seizures are widespread globally.^[27]^

Our findings have important public health and clinical implications, particularly for Saudi Arabia. There is a pressing need for targeted educational campaigns focusing on demographics with lower awareness, such as men, younger adults, and nonmedical populations. Educational materials should address specific gaps identified here, like seizure first aid and less recognized stroke symptoms such as sudden headaches. Given social media’s dominant role as an information source, platforms like Twitter, WhatsApp, and TikTok must be leveraged carefully to spread accurate information and counteract myths. Interactive content, including simulation videos and community leader partnerships, can increase engagement.^[9]^

Concurrently, regulating misinformation on social networks is critical to prevent false remedies from undermining public knowledge. Policymakers should integrate stroke and seizure awareness into school curricula and workplace training, as supported by high public agreement in various surveys. Healthcare providers and institutions must also increase community outreach via public workshops or health fairs timed around awareness days. Improving bystander recognition and response is vital; modeling studies indicate that timely hospital arrival can substantially increase treatment rates and improve stroke outcomes. Similarly, proper seizure first aid can prevent injuries and complications. Investing in public education now could reduce neurological emergency morbidity and mortality in Saudi Arabia, where stroke burden is rising and epilepsy remains prevalent.

Strengths of our study include a large, diverse sample from multiple Saudi regions and a validated questionnaire covering stroke, seizures, and SAH, providing a broad perspective on public awareness. Limitations include convenience sampling through online surveys, which may bias towards educated respondents and exclude those without internet access. Self-reported knowledge can also be overestimated or affected by recall bias. Therefore, our results may be somewhat optimistic. Nonetheless, findings align with other regional studies and offer valuable insights. Future research should consider random sampling and in-person assessments to validate public knowledge further. We acknowledge that a significant proportion of our respondents (approximately 80%) were aged 18 to 30 years. This demographic skew suggests that our findings primarily reflect the knowledge and awareness levels of young adults. Consequently, caution should be exercised when generalizing these results to the broader Saudi population, which includes a wider age spectrum. Future research should aim to include a more diverse age group to better assess knowledge levels across different age cohorts and improve the generalizability of the findings. We included stroke, epilepsy, and SAH in our study because, despite its lower prevalence, SAH is a life-threatening emergency with high morbidity and mortality. Assessing awareness of these conditions provides a comprehensive view of public knowledge and readiness to respond to critical neurological events.

In summary, public awareness of neurological emergencies in Saudi Arabia remains insufficient and uneven. Large segments of the population fail to recognize stroke or seizure symptoms or understand how to respond appropriately, potentially delaying life-saving care. Our findings reinforce the urgent need for enhanced public education and outreach. Implementing targeted awareness campaigns and embedding emergency recognition training into communities will empower citizens to act swiftly during neurological crises. Improving public knowledge is critical for ensuring timely treatment and better outcomes.

## 5. Conclusion

This cross-sectional study revealed moderate but insufficient knowledge of neurological emergencies among the Saudi population. Key findings include significantly higher awareness among females compared to males, healthcare professionals versus non-healthcare workers, and regional variations in knowledge levels. Despite general recognition of stroke as an emergency, detailed symptom recognition and proper seizure first-aid knowledge remained limited. Social media emerged as the primary information source, though often providing incomplete or inaccurate guidance. We recommend implementing targeted educational campaigns via social media platforms, and encouraging healthcare provider participation in community outreach. These initiatives should address specific knowledge gaps identified in this study while considering regional and cultural factors to ultimately reduce morbidity and mortality from neurological emergencies in Saudi Arabia.

## Author contributions

**Conceptualization:** Shorog Althubait, Layan Saeed Alshmrani.

**Data curation:** Wrod Ahmed S. Alqahtani, Mohammed Abdullah Alzayd, Mohammed Moteb M. Alshahrani, Shahad Ali M. Alshehri, Saud Turki M. Alqahtani.

**Funding acquisition:** Syed Esam Mahmood.

**Investigation:** Shorog Althubait, Taif Ali A. Almazni, Ghaida Mohammed Al Hunaif, Layan Saeed Alshmrani, Wrod Ahmed S. Alqahtani, Mohammed Abdullah Alzayd, Mohammed Moteb M. Alshahrani, Shahad Ali M. Alshehri, Saud Turki M. Alqahtani, Layan Dulaym Dashnan.

**Methodology:** Shorog Althubait, Taif Ali A. Almazni, Ghaida Mohammed Al Hunaif, Layan Saeed Alshmrani, Shahad Ali M. Alshehri, Saud Turki M. Alqahtani, Layan Dulaym Dashnan.

**Project administration:** Shorog Althubait.

**Supervision:** Shorog Althubait.

**Validation:** Taif Ali A. Almazni, Ghaida Mohammed Al Hunaif, Layan Saeed Alshmrani, Layan Dulaym Dashnan.

**Visualization:** Taif Ali A. Almazni, Ghaida Mohammed Al Hunaif, Layan Saeed Alshmrani.

**Writing – original draft:** Shorog Althubait.

**Writing – review & editing:** Syed Esam Mahmood.
